# The Foreign Journals: Anatomy and Physiology

**Published:** 1842-01

**Authors:** 


### PART THIRD.

### Selections from the British and Foreign Journals.

---

### I. THE FOREIGN JOURNALS.

---

### ANATOMY AND PHYSIOLOGY.

*On the Motions of Respiration, with especial regard to Neurological Questions.*
By A. W. VOLKMANN.

BRACHET and many with him maintain that we breathe because the sensation of the necessity of doing so instigates us; and that this necessity we feel by means of the vagus nerve. His proof is that after division of the vagus this sensation ceases; the animals do indeed breathe after the operation, but it is only by habit, and when they at last die of suffocation they do not show the least sign of agony or desire of breath. But there are many objections to this doctrine. Habit ought not to be able to maintain the motions of respiration more than those of any other apparatus whose excito-motory nerves are divided; and rabbits five hours old will breathe after their vagi are cut, though surely not from habit. Besides, it is not probable that a sensation of the necessity of breathing would depend on the lungs alone, since when breathing is stopped all the body together suffers. And direct experiments prove Brachet's opinions false.* The author repeatedly fastened stop-cocks to the tracheæ of animals, in such a manner that he could at pleasure supply them with air or cut off the supply, and in every instance the signs of agony when the access of air to the lungs was prevented, were equally intense whether the vagi nerves had been divided or not. It was obvious therefore that the sensation of a need of breathing was not centripetally conducted through the vagus.

Marshall Hall's view is that the respiratory movements are partly reflex and partly voluntary. The former, he holds, are induced chiefly by the carbonic acid which excites the branches of the vagi in the lungs, and, on some occasions, by cold applied to the skin. But if the vagi be divided, he thinks we breathe voluntarily, because we feel the need of breathing, which is conveyed centripetally not by the vagi but by other nerves; and if both the vagi be divided, and the brain removed, then the respiratory movements cease altogether, because both the centripetal conduction through the vagi, and the centrifugal impression of the will, are prevented. But, the author holds, that this theory will not agree with the law observed by Davy and Arnold that animals breathe more slowly directly after the division of the vagi than they did before; nor with the fact that in numerous experiments he found young animals (dogs, cats, and rabbits) continue to breathe for a time after division of the vagi, and complete removal of the cerebrum and cerebellum.

Seeing then, that the involuntary motions of respiration do not depend exclusively on the vagus, the question comes, what nerves are there besides the vagi,

* Dr. Volkmann mentions other errors of this author sufficient to induce much caution in receiving both his statements and his opinions.

which excite the medulla oblongata to reflex action? Are they the pulmonary branches of the sympathetic? To determine this, the author removed from a young cat, the brain, (with the exception of the medulla oblongata,) divided the nervi vagi, and, leaving the phrenic nerves, extirpated the lungs. But breathing still went on for forty minutes! all its movements continued energetic; the diaphragm was powerfully contracted, and the thorax fully elevated. The same experiment repeated in several young dogs produced the same results.

Other authors, again, regard the medulla oblongata as the *primum mobile* of the respiratory movements; thinking that it is its peculiar office to keep them in action; and that for this it does not require a special stimulus, but only the co-operation of their general forces which are indispensable for the maintenance of vitality. But this view will in no degree explain the first respiration after birth; for we should have to suppose that the medulla, which is sufficiently well developed in the uterus for the production of other movements, is suddenly charged with vital power for these particular movements directly after birth; a supposition which is most improbable.

It therefore still remains to find what is the stimulus which first excites the medulla oblongata, (the centre of the respiratory movements,) and which in after-life still continues to excite it and through it the respiratory motions. It is not either the carbonic acid in the lungs, or the atmospheric air, for the former cannot exist in quantity sufficient to have influence till respiration has commenced, and, for the latter, land animals breathe even when they are born in water or in their fœtal membranes; a fact of which the author and Dr. Schneider, confirming Leclard's observations, have frequently convinced themselves. The author believes, therefore, that the stimulus by which the reflex motions of respiration are brought on and continued, is the carbonic acid, not that which is set free in the air-passages, but that which is in the blood, that the part in which it excites is every portion of the body, and not merely the mucous membrane of the lungs; and that the excitant nerve is any nerve with a power of centripetal conduction and of action on the medulla oblongata, and not exclusively the nervous vagus.

The following therefore is, in brief, his doctrine. The respiratory motion finds its impulse in the need of respiration, and this need arises from certain relations of nutrition, not of particular parts, but of the whole body. All parts receive oxygen from the blood for the carbonic acid which they give to it; and as soon as the blood surcharged with carbonic acid cannot satisfy this need, an unnatural state of the part ensues which may be regarded as the organs' *want of breath* (Athemnoth.) This want of breath exists constantly in a minimum, because the animal substance is constantly tending to oxygenization, even when the lungs are properly in action. In this minimum condition it acts on the centripetal nerves, and through these on the spinal marrow, without, however, exciting any sensation; but when the same need rises to an excess, a sensation of it is not wanting, and it gives rise to the sense of suffocation. In all this there is nothing assumed but the exciting influence of animal tissues needing respiration on the nerves, and this is no violent hypothesis.

The theory thus proposed is in every respect sufficient for the explanation of former obscurities, if only it can be proved that a great number of the centripetal nerves are capable of so exciting the medulla oblongata as by a reflex influence to bring on the respiratory movements. And this is to a considerable extent already proved. The influence of mechanical irritation of the air-passages in beheaded animals, of the sudden application of cold to the skin, and of mechanical injuries of it in young animals, and of stimulating the cervical and other nerves, and the intimate relation between morbid conditions of various organs and changes in the mode of respiration, all tend to prove that these movements of breathing are affected by the state of the nerves of many other parts besides the lungs.

It is, moreover, in favour of this theory that by it alone the following facts are explicable: 1. That the fœtus does not breathe before birth, although it has attained all the development sufficient for the respiratory movements; the reason being, that there exists no want of breath, because the umbilical veins convey as much oxygen to the parts as they require. 2. That the fœtus does breathe directly after birth, though it comes into the world in an irrespirable medium, such as water, because after division of the umbilical vessels, want of breath ensues. 3. That every separate portion of the body which is interested in the respiratory movements, so long as it remains connected with the living medulla oblongata, continues its respiratory action, although separated from the rest of the general apparatus: in a young dog, the cerebellum and cerebrum were removed and the medulla divided at the first cervical vertebra, and yet the larynx continued its natural respiratory actions. 4. That under almost every condition of life the number of respirations and pulsations increases and diminishes in a certain parallelism, for the number of the heart's beats is a kind of numerical expression for the energy of nutrition, and if nutrition is going on rapidly, the want of breath (in the sense assumed by the author,) is proportionally increased.

[The above experiments made by Volkmann are similar to, and the results he has arrived at are exactly the same as those made by Dr. John Reid, and published some time ago in the Edinburgh Medical Journal, (Nos. 134 and 139,) but which appear to be unknown to Volkmann. In these memoirs Dr. Reid adduces numerous experiments to show that the *besoin de respirer* continues after section of the pneumogastric nerves, and shows the insufficiency of the statements of Brachet on this subject. Dr. Reid also proved that the respiratory movements continued after removal of the cerebrum and cerebellum, and insists that the inferences from these experiments are subversive of the doctrines entertained by Dr. Marshall Hall on this subject. Dr. Reid also adduces experiments to show that the respiratory muscular movements will continue after the sympathetic nerve is divided and the lungs removed; so that the experiments of Volkmann are only of importance, that they confirm in every respect these previously performed and published by Dr. Reid, and that apparently in perfect ignorance of what had been done by him. Volkmann throws out a suggestion which is not contained in Dr. Reid's memoirs, viz. that the want of oxygen in the blood and its being surcharged with carbonic acid, may act as an excitant upon the incident nerves of the body generally, and so produce respiratory movements; while Dr. Reid gives, as a probable explanation of the continuance of the respiratory movements after section of the vagi and removal of the lungs, the impressions made upon the filaments of the fifth pair and the spinal nerves distributed in the skin. It should be remembered that the respiratory movements are much diminished in frequency after section of the vagi, falling, as Dr. Reid found in his experiments, to about one half their former frequency. The experiments adduced by Volkmann in favour of his suggestion of the cause of the continuance of the respiratory movements after section of the vagi nerves, do not by any means justify this inference; for it is perfectly obvious that if the lungs were removed and the vessels left united, all the blood of the body would, in a very short time, escape from the blood-vessels; or if the pulmonary vessels were tied up it is equally obvious that the circulation would soon come to a stand; yet we are informed that the respiration continued in young animals for forty minutes after extirpation of the lungs, cerebrum and cerebellum, and section of the vagi. We have also ourselves reason to know that the respiratory movements will continue for a short time after the vessels are drained of their blood, and consequently cannot entirely, if at all, be explained by the theory of Volkmann. We are not acquainted with any experiments made by Davy on the effects of the division of the vagi upon the respiratory movements, and strongly suspect that Volkmann has here fallen into some mistake.]

*Müller's Archiv.* Heft iv. 1841.

*Experiments on Muscular Irritability and Nervous Action.* By M. Longet.

The object of M. Longet is to determine the degree or limit to which muscular irritability is subjected to nervous action. At the sitting of the Royal Academy of Sciences, May 24th, 1841, he proved that a motor nerve, which has been separated from the brain or spinal marrow, loses on the fourth day its power of producing contraction of the voluntary muscles, under the influence of a stimulus, even that of galvanism. In a second communication at the sitting on the 26th July, he brought forward his researches on the question whether muscular irritability disappeared or not with nervous excitability; which were instituted because he thought that Haller's experiment to prove that there was an inherent power of contraction in muscular fibre independent of nervous force was fallacious, contraction of the heart persisting after its having been torn from the chest by virtue of latent nervous power. To avoid this source of error he divided the sciatic nerve and applied galvanism four days afterwards to its free extremity without exciting the least trembling of the muscles to which it was distributed; and yet at the end of fifteen days these muscles contracted strongly under the influence of a very slight stimulus *immediately* applied. After a month the power of contraction although less is still very sensible, and it was appreciable towards the seventh week. At that time the muscular fibre had degenerated and ceased to contract under the immediate application of the strongest stimulants. It is only, then, in consequence of a lesion of nutrition, of which the effects are slowly pronounced, that the muscular fibre in losing its organic characters, loses also its essential property, irritability, which exists long after all nervous influence has ceased.

[Though we think it right to give insertion to the above in order to show what our neighbours are doing in physiology, we must remark that Dr. John Reid has already *proved* all that is here indicated. In addition to what is here stated, Dr. Reid has shown that the irritability of the muscle after section of the nerve may be maintained by its frequent exercise, and that it may be recovered, if exhausted, provided the nutrition of the muscle be not impaired.]

*L'Examinateur Médical. Juillet* 25, 1841.

---

*Experimental Researches on the Functions of the Nerves of the Larynx, and on the Influence of the Accessory Nerve of Willis on Phonation.* By M. Longet.

This is a very able and important paper, but so exceedingly long that we can only give the conclusions at which the author arrives as the result of his experiments.

1. The superior and inferior laryngeal nerves have influence upon phonation.

2. Of the two branches furnished by the superior laryngeal, the external only (which supplies the crico-thyroid muscle), when it is divided, modifies the voice; and the internal does not govern the contractions of the arytenoid muscle, but the sensibility of the laryngeal mucous membrane only.

3. If the nervous filaments of the crico-thyroidei be divided alone, or in other words if these muscles be paralysed, the voice becomes disagreeably hoarse, and their paralysis prevents the production of cries in young animals (which after division of the recurrents still utter acute cries), at the same time that it augments the difficulty of respiration.

4. After division of the recurrents very young dogs still produce sounds remarkable for their acuteness, although older dogs remain dumb; these facts have been explained by the special conformation of the vocal and respiratory parts of the glottis, parts the relative dimensions of which vary with age.

5. These differences in the relative size of these portions, in the form and density of the arytenoid cartilages at different ages, may explain the danger of imminent suffocation at one age ceasing at the other.

6. Animals deprived of their inferior laryngeal nerves respire more quickly than in the normal state.

7. The contraction of the arytenoideus is subjected to the recurrent and not to the superior laryngeal; this muscle is the especial *constrictor of the respiratory glottis.*

8. The lateral crico-arytenoid muscles, also supplied by the recurrent, do not dilate the glottis as most physiologists assert; on the contrary they contract it and are the especial *constrictors of the vocal glottis.*

9. It is therefore incorrect to state that the constrictor muscles of the glottis receive their nerves from the superior laryngeal, and that after the section of the recurrent nerves, these muscles preserve their action, closing the glottis.

10. The pneumogastric governs the sensibility of the larynx, the intrinsic movements of which are subordinate to the accessory nerve of Willis.

[In a large part of his conclusions in this paper also—all, in fact, which concerns the special functions of the *superior* and *recurrent* laryngeal nerves—the author has been anticipated by Dr. J. Reid, who has also noticed and explained the difference between young and old dogs, in regard to the effects of section of the nerves on respiration. The only points absolutely new are the statements that the arytenoidei are the constrictors of the *respiratory glottis,* and the *crico-arytenoidei* of the vocal glottis. The inference No. 10 necessarily follows from Valentin's doctrine that *all* the motor fibres of the pneumogastric are derived from the spinal accessory; these two nerves being, at their origins, analogous to the two fasciculi of roots of the spinal nerves. (See Br. and For. Med. Rev., vol. XI. p. 291.)]

*Gazette Médicale de Paris. Juillet* 24, 1841.

---

### *On the Functions of the Epiglottis, and on the Closure of the Glottis in Deglutition, &c.* By M. LONGET.

THE author remarks that four causes are opposed to the introduction of solid or liquid food into the air-passages; 1st, The movement of the larynx upwards and forwards combined with that of the tongue backwards whose base is applied on the upper orifice of the larynx; 2d, The epiglottis, which, placed between the larynx and the base of the tongue, follows the movement given to it by the latter, and, as it were, moulds itself upon the glottis; 3d, The exquisite sensibility of the mucous membrane covering the supra-glottic space; 4th, The occlusion of the glottis.

A remarkable fact pointed out by M. Longet is that in the second period of deglutition, the closure of the glottis is still effected after the division of the nerves which govern all the proper muscles of the larynx. He saw that this is due not to the crico-thyroid muscles which are thus paralysed, nor to the thyro-hyoid (which he removed), but to the inferior constrictors of the pharynx, which, embracing the diverging alæ of the thyroid cartilage, folds them one against the other, approximating the borders of the glottis, and pressing on the muscles external to it.

The epiglottis was completely excised in six dogs; solid food still passed with facility, but the deglutition of liquids was often not accompanied but followed by a convulsive cough, which the author explains by the falling into the supra-glottic vestibule of some drops of liquid, which even after deglutition still lie upon the dorsum of the tongue, but which, if the epiglottis is in its place, flow over it and over the back part of the larynx. The removal of the epiglottis did not materially affect the voice.

If, in a dog, one cuts the two recurrent nerves and the branches supplying the crico-thyroid muscles, so as to paralyse all the proper muscles of the larynx, but leaves the internal laryngeal nerve, which presides over the supra-glottic sensibility, intact, and then makes the animal drink cautiously, nothing passes into the trachea. But if the internal laryngeal nerves be also divided, the glottis still closes, but some drops of fluid fall into the trachea, because the animal not being aware in time of the presence of the liquid, its glottis often closes too late.

The occlusion of the glottis is not indispensable to the deglutition of liquids poured cautiously; for M. Longet has made animals swallow though he kept the borders of the glottis a little separated, by means of dissecting forceps.

He concludes that the displacement of the base of the tongue and the epiglottis are the two most important conditions, and that the closed glottis is only the last barrier which nature sets up against the passage of food into the larynx.

*L'Examinateur Médical. Octobre* 17, 1841.

---

*On the Occurrence of Urea in the Blood.* By J. F. Simon.

The author has never failed to find urea in the blood of those who have died with the granular degeneration of the kidneys. In the blood also of a woman who died with all the signs of cholera, he found a very large quantity; one sufficient for him to obtain crystals of pure urea in very long quadrilateral prisms visible even to the naked eye. This same blood contained a remarkable quantity of biliverdine and biline, so that its taste was strongly bitter. He has lately determined that healthy blood contains a very small quantity of urea; from about sixteen pounds of calf's blood treated by a lengthened, but apparently very accurate process, he obtained distinct crystals of nitrate of urea, but not a trace of biliary matter.

*Muller's Archiv.* Heft v. 1841.

---

*A Case of Double Vagina, Double Cervix Uteri, and, probably, Double Uterus.* By Dr. Fricke, of Hamburg.

A girl, twenty-one years old, was received into the venereal ward of the hospital, on December 17, 1840. She had once before been under medical treatment for gonorrhœa and bubo, and had for three years been leading an unchaste life. Sexual intercourse was not attended with any particular difficulty, and she was wholly unaware of the peculiarities of her generative organs.

The condition of the labia was natural, the left nympha was hypertrophied and projected between them, while the right was of the ordinary size. On separating the nymphæ the orifice of the vagina was seen to be divided by a septum arising on either side and from the posterior surface of the urethra, and being attached to the posterior surface of the vagina a little to the right of the mesial line. Its breadth at its origin was about one inch and a quarter, it then narrowed to half an inch, again expanding to an inch in breadth at its insertion. This septum was found to extend up the vagina, and to be about two lines in thickness. It was not perpendicular, but curved with its convexity to the left, and the right vagina was that which, according to the woman's statement, had been always used in sexual intercourse. The left vagina was narrower than the right, and the os uteri at its termination appeared smaller when viewed with a speculum, but well-formed and resembling that on the right side. A sound could be introduced into each os uteri, to the depth of three-quarters of an inch on the right side, and of one inch and a half on the left side; but this depth was not sufficient to allow of bringing the ends of the two sounds into contact, and of thus ascertaining whether the uterus was likewise divided by a septum, or whether each os uteri terminated in a common cavity.

On examining *per anum,* a depression could be felt extending for an inch between each cervix uteri; but beyond this point the finger could not reach.

The patient had not menstruated since her admission into the hospital, it was therefore not possible to say whether the secretion flowed from both, or only from one os uteri.

*Zeitschrift für die gesammte Medicin. October,* 1841.

---

*On a Ligament in the Velum Palati.* By Dr. Pappenheim.

It is easy to see with the naked eye that there is a white streak on the anterior median line of the velum, which takes its origin from the lowest extremity of the uvula, thence ascends, dividing the velum into two equal halves, and proceeds to the membrane of the hard palate, where, though it grows thinner, it may be traced to the upper incisor tooth. On the posterior median line of the velum this streak, though less distinct, yet in like manner proceeds from the uvula to the posterior nasal spine. The microscope shows that it consists of large and chiefly longitudinal fasciculi of strong elastic fibres, which are traversed by only a few blood vessels and nerves, and which spread out on either side of the median line of the velum, beneath its mucous membrane, towards the tonsils. It is observable that the middle portion is favorable to the considerable power of contraction which the uvula possesses in the longitudinal direction, and the lateral fibres to the mobility of the whole velum.

The streak to which, only for the sake of indicating its independent existence, Dr. Pappenheim gives the name of ligamentum uvulæ, occurs as well in tender children as in adults, and sometimes splits anteriorly into two halves.

*Medicinische Zeitung. Juli* 14, 1841.

---

*On Animal Heat.* By MM. BECQUEREL and BRESCHET.

THE experiments of M. Fourcault has shown that if the cutaneous exhalation from any animal be prevented by covering its body with some substance impermeable to watery vapour, it dies ere long with many of the signs of ordinary asphyxia. The authors, regarding the exhalation as one of the chief means by which the body loses heat, supposed that by checking it in this perfect manner, a kind of fever would be produced, and the temperature of the animal's interior would be greatly increased. They tried this with delicate and well-managed thermo-electric apparatus, and the result was the very opposite of that which they expected. Of many experiments they relate two, made on rabbits. In one, within half an hour of his being enveloped with the impermeable varnish, the temperature of the deep muscles fell from 38° to 32°; and in half an hour more to 24° 5′. In the other, after half an hour, the temperature of the muscles was only 3° above that of the surrounding medium, which at that time was 17°; it had therefore fallen in this short time 18°. In an hour and a half the animal died.

With similar apparatus, the authors confirmed the facts which they and many others had already ascertained, of the temperature of arterial being higher than that of venous blood. In one of their experiments, on a dog, they found the temperature of the blood in the right auricle 37° 5′; of that in the left auricle 38°·05; showing therefore an increase equal to 0°·65 in the passage of the blood through the lungs.

*L'Examinateur Médical. Octobre* 24, 1841.

---

*On the Production of the Blood-globules.* By Dr. REMAK, of Berlin.

IN the blood of chicks during the third week of artificial incubation I found blood-corpuscles, some of which were round, some pear-shaped and stalked, and some *biscuit*-shaped, with their large ends coloured red, and containing each a nucleus. These two nuclei were connected together by a thin process which traversed the uncoloured and canal-shaped intermediate portion of the corpuscle. The nuclei of the stalked corpuscles also exhibited a process corresponding to the peduncle of the corpuscle. These observations therefore render it probable that in these instances an increase of the corpuscles is effected by means of *division.* The corpuscles of the embryos of pigs an inch long were from four to six times larger than those of adult pigs; they exhibited double or quadruple nuclei, which probably belonged to different divisions of the blood-corpuscle marked by pale intermediate lines.

To observe the reproduction of blood-corpuscles after losses of blood, thirty pounds of blood were drawn from a horse; and a part of it when examined was found to contain, besides the well-known corpuscles without nuclei, only a few pale lymph-corpuscles, as they are called. On the following day the latter were found in enormous quantities and much enlarged; and in their interior they exhibited one or more pale reddish globules of the size of blood-corpuscles, covered by the granular contents. On succeeding days these globules appeared the more red the more the granular contents of the parent-cells (for such the pale lymph-corpuscles proved to be) diminished, and the thinner their membrane became. On the fourth day there could be no doubt that the red blood-corpuscles form within the enlarged pale cells, and become free by the disappearance of the latter. The blood of the horse became, as further experiments showed, the more coagulable, and the thickness of the buffy coat became the greater the more blood was drawn ; but the buffy coat in such a case consisted of but little coagulated fibrine, with an excessive quantity of the parent-cells of blood-globules.

I have confirmed these results in man about forty times Between the fourth and eighth days after the first considerable abstraction of blood, even during inflammatory and typhous diseases, the commencing regeneration of blood-corpuscles is seen in the appearance of parent-cells, which, as in horses, in consequence of their low specific gravity, are chiefly found in the coagulum of the buffy coat, of which indeed they form a considerable part. My present investigations lead me to expect that I shall succeed in finding easily discernible physical differences between the buffy coats formed by an excess of parent-cells, and those which consist chiefly of coagulated fibrine, and which are the results of the great specific gravity of the blood-corpuscles causing them to sink in slowly coagulating blood. I shall at present only say, that the looseness of the buffy coat is generally the consequence of an excess of new parent-cells. A buffy coat which appears five days, or perhaps even a shorter time after the first bleeding, can never be a true sign of inflammation.

With respect to the production of the pale parent-cells, my present investigations render it probable that they are generated, not within the blood, but in the cells which line the walls of the blood-vessels and lymphatics, but experiments in which I am now occupied will determine this.

*Medicinische Zeitung. Juli* 7, 1841.

---

*Absence of the Uterus.* By Dr. Cramer.

The case here related is that of a woman 30 years old, of perfectly feminine external form, who had never menstruated but had frequently suffered from epistaxis, hæmoptysis, and other signs of local congestions. She married at 23, and at 30, annoyed at having no children, submitted to an examination. No uterus could be found, but the external organs of generation were well formed, and the vagina was of normal width, as long as the finger, and ended in a cul-de-sac. The unusual circumstances of the case were, the existence of natural sexual appetite, and of a tendency to vicarious menstruation, the normal development of the external sexual characters, and the nearly full size of the vagina; all rendering it probable that, though the uterus was deficient, the ovaries were in their natural state.

*Medicinische Zeitung. August* 18, 1841.

